# Rough Titanium Oxide Coating Prepared by Micro-Arc Oxidation Causes Down-Regulation of *hTERT* Expression, Molecular Presentation, and Cytokine Secretion in Tumor Jurkat T Cells

**DOI:** 10.3390/ma11030360

**Published:** 2018-02-28

**Authors:** Igor Khlusov, Larisa Litvinova, Valeria Shupletsova, Olga Khaziakhmatova, Elena Melashchenko, Kristina Yurova, Vladimir Leitsin, Marina Khlusova, Vladimir Pichugin, Yurii Sharkeev

**Affiliations:** 1 Department of Experimental Physics, National Research Tomsk Polytechnic University, Tomsk 634050, Russia; pichugin@tpu.ru (V.P.); sharkeev@ispms.tsc.ru (Y.S.); 2 Laboratory of Immunology and Cell Biotechnology, Immanuel Kant Baltic Federal University, Kaliningrad 236029, Russia; vshupletsova@mail.ru (V.S.); hazik36@mail.ru (O.K.); lena.melashchenko17@mail.ru (E.M.); kristina_kofanova@mail.ru (K.Y.); leitsin@mail.ru (V.L.); 3 Department of Pathophysiology, Siberian State Medical University, Tomsk 634050, Russia; uchsovet@ssmu.ru; 4 Institute of Strength Physics and Materials Science of SB RAS, Tomsk 634055, Russia

**Keywords:** titanium substrate, surface electrostatic potential, TiO_2_ nanoparticles, tumor cell death, reactive oxygen species, in vitro

## Abstract

The response of the human Jurkat T cell leukemia-derived cell line (Jurkat T cells) after 24 h of in vitro exposure to a titanium substrate (12 × 12 × 1 mm^3^) with a bilateral rough (*R_a_* = 2.2–3.7 μm) titanium oxide coating (rTOC) applied using the micro-arc method in a 20% orthophosphoric acid solution was studied. A 1.5-fold down-regulation of hTERT mRNA expression and decreases in CD3, CD4, CD8, and CD95 presentation and IL-4 and TNFα secretion were observed. Jurkat T cell inactivation was not correlated with the generation of intracellular reactive oxygen species (ROS) and was not mediated by TiO_2_ nanoparticles with a diameter of 14 ± 8 nm at doses of 1 mg/L or 10 mg/L. The inhibitory effect of the rTOC (*R_a_* = 2.2–3.7 μm) on the survival of Jurkat T cells (Spearman’s coefficient *r_s_* = −0.95; *n* = 9; *p* < 0.0001) was demonstrated by an increase in the necrotic cell count among the cell population. In turn, an elevation of the Ra index of the rTOC was accompanied by a linear increase (*r* = 0.6; *p* < 0.000001, *n* = 60) in the magnitude of the negative electrostatic potential of the titanium oxide surface. Thus, the roughness of the rTOC induces an electrostatic potential and decreases the viability of the immortalized Jurkat T cells through mechanisms unrelated to ROS generation. This may be useful for replacement surgery applications of rough TiO_2_ implants in cancer patients.

## 1. Introduction

The highly proliferative Jurkat line of human leukemic T lymphoblast-like cells (Jurkat T cells) is widely used in toxicological research for the in vitro modeling of normal blood Т lymphocyte reactions [1] and of the cells that are involved in acute T lymphoblastic leukemia and lymphoma [2]. Because of largely similar immunophenotype and cytokine profile with human blood CD45CD3^+^ primary cells, Jurkat T cells are actively used to study immune and cytotoxic reactions to anticancer drugs [3] toxicants [4], and biomaterials [5], as well as ions [6] and titanium dioxide nanoparticles [7]. The last two substrates are released during the biodegradation of endoprostheses and during triggered immunopathological reactions.

Titanium is a modern biomaterial with bio-inert properties that is broadly used in various areas of biomedicine [8], primarily due to the ability of titanium dioxide to form surface metal-ceramic films [9]. Recent publications provide evidence of the physicochemical properties of Ti surfaces transcending the bio-inert concept [10]. Titanium dioxide is very insoluble, and biologically stable. It cannot pass undamaged skin, and even when inhaled or ingested, TiO_2_ is not thought to have serious effects on humans. Moreover, zinc doped-titanium oxide nanoparticles are thought to be promising candidates for therapeutic angiogenic applications in cases of cardiovascular pathology [11]. However, there are also reports indicating that TiO_2_ particles may be considered a biohazard [7]. 

For instance, there is a yet unsolved problem (up to partial or complete destruction) related to wear particles produced by implants and prostheses. The latter manifests itself in the accumulation of wear debris (ions, nanoparticles) in tissues and their massive escape from tissues into blood [12,13], with continual release of particles of titanium alloys into the tissues [14]. The generated metal particle (wear debris) sizes range between 40 and 50 nm [12] and are distributed throughout the vascular system [15]. 

For all this, blood concentrations of metal wear debris may reach the toxic value of 6.5 mg/L to induce systemic wear-related complications [16]. The particles remain in the soft tissues; local concentrations of titanium in patients may be 304 mg/kg of dry tissue [17]. Therefore, the experimental research of the oxide metal nanoparticles toxicity in vitro is very significant for spine and dental implant surgery, orthopedics, and traumatology. 

Furthermore, Curtis and Varde [18] hypothesized that the topography of natural and artificial matrices plays a crucial role in determining cell behavior. Indeed, various types of cells, including Jurkat T cells [19], react directly to surface micro- [20] and nanotopography [21].

Indeed, a series of works focused on determination of the relation between either smooth or grooved titanium and its alloys surface roughness and adhesion [22] or proliferation [23] of adherent cells, mainly osteoblasts. A close relation between the surface roughness amplitude and cell adhesion was not found. As a result, a new parameter, called the “adhesion power”, was developed [24]. However, the underlying processes and potential mechanisms regulating tumor cell reactions on rough metal surface of implants and endoprosthesis remain insufficiently investigated.

This study aimed to examine the morphofunctional reaction of Jurkat T cells to short-term in vitro exposure to a rough titanium oxide coating and their possible wear debris, such as TiO_2_ nanoparticles. 

## 2. Materials and Methods

### 2.1. Substrate Preparation and Coating Deposition and Characterization

Commercially pure titanium (99.58 Ti, 0.12 O, 0.18 Fe, 0.07 C, 0.04 N, 0.01 H wt. %) plates (12 × 12 × 1 mm^3^) were used as substrates for the deposition of titanium oxide coatings. The samples were cleaned ultrasonically with an Elmasonic S10 (Elma Schmidbauer GmbH, Sigen, Germany) for 10 min in distilled water immediately before deposition. The bilateral coating was prepared according to the anodal regime as described previously [25] using a Micro-Arc 3.0 apparatus (Institute of Strength Physics and Materials Science of SB RAS, Tomsk, Russia). The setup consists of a pulse power source, a computer to control the deposition process, a galvanic bath with water cooling, and the electrodes. An aqueous solution of 20% phosphoric acid was employed to obtain the coating. The coating was formed in pulse mode with the following parameters: pulse time, 100 µs; pulse frequency, 100 Hz; deposition time, 20 min; voltage, 250 V. The specimens were dried using dry heat with a Binder FD53 (Binder GmbH, Tuttlingen, Germany) at 453 K for 1 h.

The surface roughness of the substrate was assessed with a Talysurf 5-120 profilometer (Taylor Hobson Ltd., Leicester, UK). Ten randomly selected traces were recorded for each specimen. The average roughness (*R_a_*), peak-to-valley roughness (*R_z_*) and maximum roughness (*R_max_*) were estimated. A strong linear correlation (*r* = 0.95; significance 99%) was identified between *R_a_*, *R_z_* and *R_max_*. Therefore, only *R_a_* was used for further roughness characterization. Samples with an *R_a_* of 1.5–4 μm were used. To obtain samples with an *R_a_* > 2 μm, the substrate surface was pretreated by Al_2_O_3_ sandblasting (particle diameter 250–380 μm, HITK, Dresden, Germany) and chemical etching. The chemical etching in acid etch on the basis of solutions of 30% hydrochloric and 60% sulfuric acids heated to a boiling temperature allows for the titanium surface to be clearer of the abrasive powder and generates multilevel surfaces.

The coating thicknesses of five witness samples were measured before and after the coating deposition (GOST 9.302-88 ESZKS) using a Russian-produced MK-25 micrometer (Micron manufactory, Moscow, Russia). The average thickness was 9 ± 2 μm.

The morphology and elemental composition of the coating surface were studied using a scanning electron microscope (SEM; Phillips SEM 515, Philips, Amsterdam, The Netherlands), equipped with an energy-dispersive X-ray spectroscope (EDAX ECON IV, Phillips, Amsterdam, The Netherlands).

**Figure 1:**
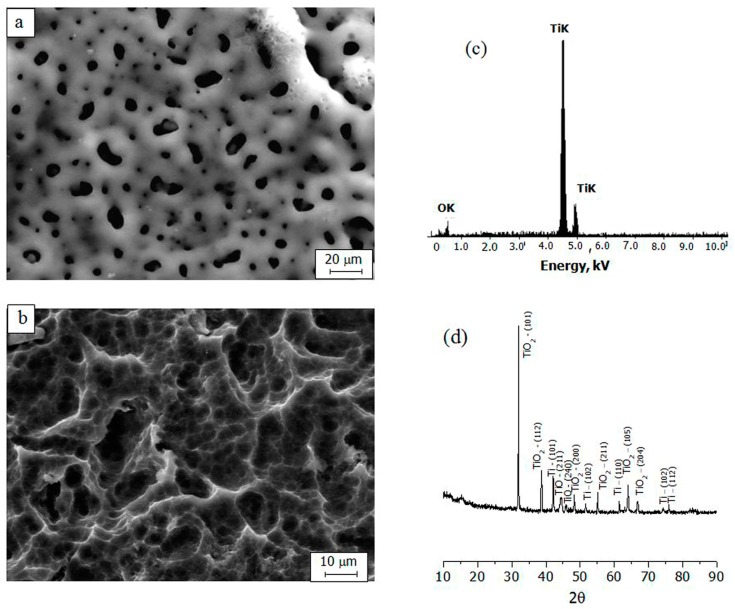
SEM-images of the titanium oxides (TiO_2_) coating before sandblasting and subsequent acid etching (**a**), the Ti surface after acid etching (**b**), EDX spectrum (**c**) and X-ray diffraction pattern of the TiO_2_ coating (**d**).

According to the SEM, the topography of the TiO_2_ coating consists of a micropore and microrough structure (Figure 1a). The size of the coating pores was measured using the standard intercept method. The general porosity was calculated as the ratio between the total length of intervals between the pores and the total length of the intercepts [25]. The surface porosity reached 20%, with an average pore diameter of 2.1 ± 0.4 μm, which corresponded with our previous results [26].

Investigation of the morphology of the titanium substrate that was subjected to sandblasting with corundum particles and chemical etching showed that the surface had a strongly pronounced relief (Figure 1b); therefore, a roughness of *R_a_* > 2 μm was reached. When TiO_2_ coating was applied to the relief of the titanium surface, groups of porous (2–5 μm in diameter) surfaces, with average *R_a_*s of 2.20 ± 0.12 μm, 2.28 ± 0.11 μm, and 3.70 ± 0.14 μm, were achieved.

The key elements of the oxide coating included oxygen (56 at %) and titanium (44 at %), according to EDX analysis (Figure 1c). X-ray diffraction analysis was performed with a DRON-7 diffractometer (Burevestnik, Nizhny Novgorod, Russia) in the angular range 2*θ* = 10–90°. The obtained diffractograms were interpreted using the International Center for Diffraction Data (ICDD) database. The phase composition of the micro-arc oxide (MAO) coating included titanium oxides (TiO and TiO_2_), predominantly TiO_2_ in the form of anatase.

An Olympus GX-71 inverted reflected light microscope (Olympus Corporation, Tokyo, Japan) equipped with an Olympus DP 70 digital camera was used to obtain dark field images of the coating relief and to locate cells.

The lifting electrode method (the Eguchi method) [27] was used to measure the surface EP on a macroscale. The measurements were conducted under ambient conditions. The homemade device has been previously described in detail [28]. This device measures the electric field potential of weakly charged bodies. The longitudinal resolution of the device was 5 mm, and the measured potentials ranged from tens of millivolts to hundreds of volts. The measuring electrode that was installed on the surface of the coating was used to measure the charge. The potential induced at the measuring electrode ($V_{in}$) is related to the surface potential ($V_{L}$) by the following expression:

(1) $$V_{L}=\frac{C_{in}+C_{l}}{C_{l}}V_{in}$$

where *C_in_* is the input capacitance of the measuring instrument and *C_l_* is the measuring capacitance.

### 2.2. Titanium Dioxide Nanoparticles

Titanium dioxide nanopowder was produced using the electric conductor explosion method at the Institute of High Current Electronics of the Siberian Branch of the Russian Academy of Sciences (Tomsk, Russia). Transmission electron microscopy (TEM) using a JEM1400 instrument (JEOL, Akishima, Japan) showed a monocentric distribution of cube-shaped particles with an average diameter of 14 nm and a standard deviation of 8 nm. The detailed features of the nanoparticles used have been described [26].

Nanoparticle suspensions were prepared immediately prior to cell loading via ultrasonic treatment (Elmasonic S10, Sigen, Germany) of a nanoparticle suspension in an isotonic sodium chloride solution for 5 min. Ten maximum allowable concentrations (MACs, 1 mg/L) or 100 MACs (10 mg/L) of nanoparticles served as the final concentrations in the cell suspension and were mixed carefully with a pipette. As a comparison test, MACs (3 mg/L) of Fe_3_O_4_ nanoparticles with an average diameter of 40 ± 23 nm were produced using the electric conductor explosion method at the Institute of High Current Electronics of the Siberian Branch of the Russian Academy of Sciences (Tomsk, Russia). The physical-chemical features of Fe_3_O_4_ nanoparticles are described in Khlusov et al. [29].

### 2.3. Cell Culture

The immortalized Jurkat 5332 cell line of human leukemic T lymphoblast-like cells that were received from the cell bank of the Institute of Cytology (Institute of Cytology, Russian Academy of Sciences, Saint Petersburg, Russia) was used at a density of 1 × 10^6^ living mononuclear cells per 1 mL of nutrient medium to assess the in vitro molecular characteristics of the TiO_2_ coating and the biocompatibility of the TiO_2_ nanoparticles. Calculations of cell concentration and viability prior to culturing were conducted with a Countess ^TM^ Automated Cell Counter (Invitrogen, Carlsbad, CA, USA) using 0.4% trypan blue solution (Invitrogen). The cultures consisted of 98% living cells.

The Jurkat T cells were resuspended in complete culture medium consisting of 90% RPMI-1640 (Sigma-Aldrich, St. Louis, MO, USA), 10% inactivated (for 30 min at 56 °С) fetal bovine serum (Sigma), and 0.3 mg/mL l-glutamine (Sigma). The wells of a 24-well test plate (Orange Scientific, Braine-l’Alleud, Belgium; well area 1.86 cm^2^) were filled with one TiO_2_-coated substrate or with a TiO_2_ nanoparticle suspension. The cell culture was then incubated for 24 h in a humidified atmosphere of 95% air and 5% CO_2_ at 37 °C. A cell suspension without three-dimensional or nanoscale samples was used as a control.

After incubation, the cell suspension was centrifuged at 500 g for 15 min. The obtained cell pellet was used to measure *hTERT* gene expression, apoptosis, necrosis, reactive oxygen species, and membrane antigen presentation. The cell culture supernatants were employed to measure cytokine concentrations. In vitro manipulation was conducted with the approval of Local Ethics Committee of Innovation Park of Immanuel Kant Baltic Federal University (Permission No. 4 from 23 October 2013).

### 2.4. hTERT Expression in Jurkat T Cells

The expression of the *hTERT* (human telomerase reverse transcriptase) gene in Jurkat T cells was analyzed via SYBR Green-based quantitative polymerase chain reaction (PCR), as previously described [30]. mRNA was extracted from Jurkat T cells using the RNeasy Mini Kit (Qiagen, Germantown Rd, MD, USA) according to the manufacturer’s instructions. Total RNA (100 ng) was subsequently reverse-transcribed into cDNA using the QuantiTect Reverse Transcription Kit (Qiagen). The thermal cycling conditions were as follows: denaturation at 94 °C for 6 s, alignment at 60 °C for 6 s, and elongation at 72 °C for 6 s (45 cycles). The *hTERT* primers are shown in Table 1. A second pair of 18S rRNA primers was used as an internal control. All of the reactions were performed in duplicate. Reaction mixtures without RNA were employed as negative controls in each run. The fold change in the expression of genes of interest was calculated using the ddCt method. The results are expressed in arbitrary units (the ratio between the relative amount of *hTERT* cDNA and the relative amount of 18S rRNA cDNA). 18S rRNA cDNA may have utility as a control for experiments using these cells, as the gene did not exhibit significant changes in expression in Malathi Banda’s study “Evaluation and validation of housekeeping genes” [31].

### 2.5. Reactive Oxygen Species and Measurement of Cell Death

**Figure 2:**
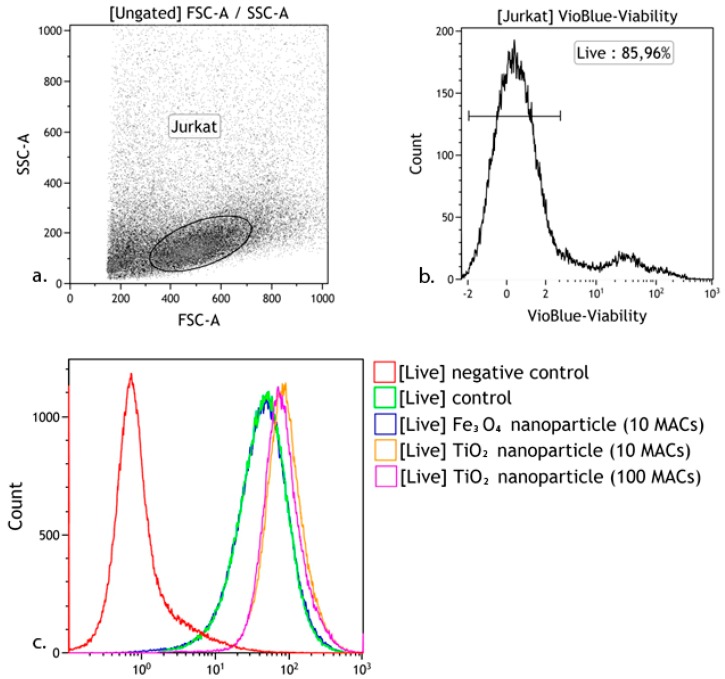
Algorithm for determining intracellular levels of ROS after 24 h in culture with the addition of a nanoparticle suspension. (**a**) Jurkat gating based on a forward scatter (FSC) vs. side scatter (SSC) plot; (**b**) The analysis was carried out using gating of Jurkat cells. The abscissa axis is the fluorescence intensity of antibodies against Viability Fixable Dye; the ordinate is the number of cells; (**c**) Evaluation of the production of reactive oxygen species.

The intracellular concentration of reactive oxygen species (ROS) and the proportion of dead cells were measured with a MACS Quant FL7 flow cytometer (Miltenyi Biotec, Bergisch Gladbach, Germany) ROS production in live cells was detected using H2DCFDA (Sigma Aldrich, St. Louis, MO, USA) (Figure 2). Levels of intracellular ROS were quantified by subtracting the background ratio of MFI (mean fluorescence intensity) to the number of cells in this gate of the non-fluorescent sample (negative control) from the same ratio of fluorescent samples in flow cytometry analysis with MACS Quant FL7 (Miltenyi Biotec): X = MFIs/ns–MFInc/nnc, (X—level of intracellular ROS, MFIs—mean fluorescence intensity of the fluorescent sample, ns—number of cells in the fluorescent sample, MFInc—mean fluorescence intensity of the negative control, and nnc—number of cells in the negative control).

**Figure 3:**
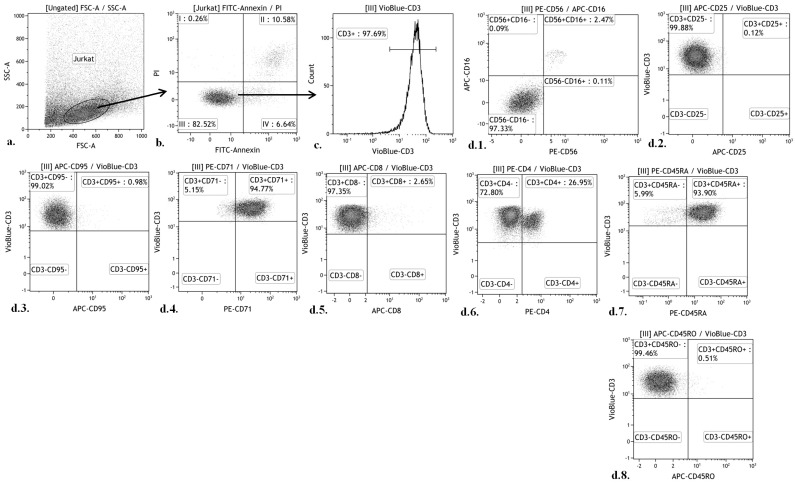
Gating strategy for Jurkat T cell CD staining. (**a**) Jurkat cell gating based on a forward scatter (FSC) vs. side scatter (SSC) plot; (**b**) The analysis was carried out using gating of Jurkat cells. The abscissa axis is the fluorescence intensity of the antibodies against Annexin; the ordinate is the fluorescence intensity of the antibodies against propidium iodide. Area “III” contains viable cells; (**c**) The analysis was carried out using gating on the “III” area. Histogram of the distribution of Jurkat cells; the axis of abscissas is the fluorescence intensity of the antibodies against CD3; the ordinate is the number of cells; (**d1**) The analysis was carried out using gating of the “III” area. Histogram of the distribution of Jurkat cells; the abscissa axis is the fluorescence intensity of the antibodies against CD56; the ordinate axis is the fluorescence intensity of the antibodies against CD16, (**d2**–**d8**) The analysis was carried out using gating of the "III" area. Histogram of the distribution of Jurkat cells; the abscissa axis is the fluorescence intensity of the antibodies against CD3; the ordinate axis is the fluorescence intensity of the antibodies against CD25, CD95, CD71, CD8, CD4, CD45RA, or CD45RO.

The ratio of living and dead (apoptotic and necrotic) cells and the overall number of cells that were used in the assay were measured via flow cytofluorometry using propidium iodide (Sigma Aldrich, USA) and Annexin V-FITC (Abcam, Cambridge, UK) with a MACS Quant FL7 (Miltenyi Biotec) (Figure 3).

### 2.6. Cytokine Profile in the Cell Culture

To measure the spontaneous secretion of interleukins (IL-1b, IL-2, IL-4, IL-6, IL-8, and IL-10) and the tumor necrosis factor alpha (TNFα) in the supernatants, a solid-phase sandwich immunoassay (SIA) was performed. The SIA procedure was conducted according to the instructions of the manufacturer of the cytokine assay system (Vektor Best, Novosibirsk, Russia) using an automated EPLISA processing system (Dynex Technologies, Chantilly, VA, USA). The concentration of each cytokine was expressed in pg/mL.

### 2.7. Cellular Immunophenotype Detection

The cellular antigen profile was analyzed using a method based on the interaction between specific monoclonal antibodies (mAbs) and clustering determinants on the cell surface, according to the manufacturer’s instructions.

After culturing, the cells were washed with phosphate-buffered saline (рН 7.2), and a single-cell suspension was mixed with a single standard mAb. Propidium iodide (Sigma Aldrich) and Annexin V-FITC (Abcam) were used to exclude dead cells, with identification using CD3-VioBlue (e-Bioscience, San Diego, CA, USA). Live Jurkat T cell subpopulations were identified using allophycocyanin (APC)-labeled anti-CD16, CD25, CD95, CD8 and anti-CD45RO (BD Pharmingen); PE-labeled anti-CD56, CD71, CD4 and anti-CD45RA (Figure 3) (Table 2).

After 30 min of incubation at 4 °C with the labeled mAb, the cells were washed in PBS, centrifuged (300× *g* for 5 min), resuspended in PBS (200 µL) and assayed using a MACS Quant FL7 (Miltenyi Biotec). The cytometric results were examined using KALUZA Analysis Software (Beckman Coulter, Brea, CA, USA).

### 2.8. Statistical Analysis

The results were analyzed using STATISTICA software for Windows 6.0. The following distribution parameters were calculated: the median (Me), the 25% quartile (Q_1_) and the 75% quartile (Q_3_). The Mann-Whitney U-test (*P_U_*) was performed, and the differences were considered significant at *p* < 0.05. The relationship between the studied parameters was established via regression and correlation (Spearman) analyses. The coefficients (*r*) were kept at a significance level greater than 95%.

## 3. Results

**Figure 4:**
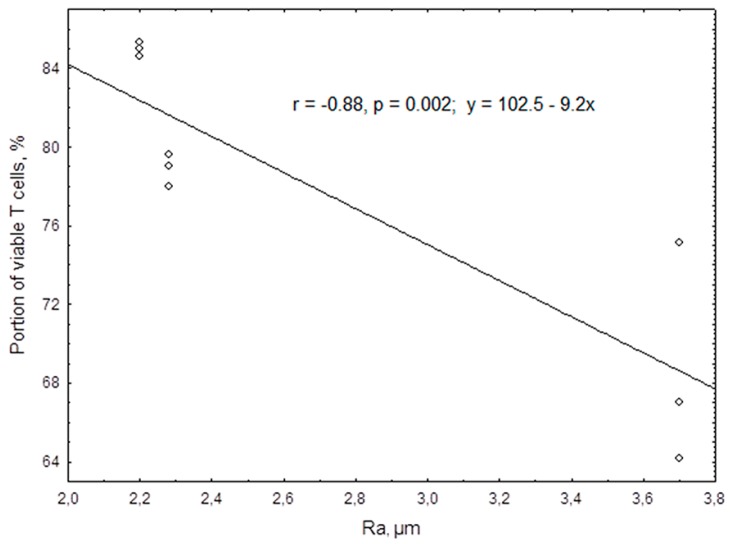
Regression of Jurkat T cell viability after 24 h of culture in the presence of rough TiO_2_-coated titanium samples.

TiO_2_-coated titanium samples did not cause an increase in apoptosis or necrosis in the selected cell system in vitro. The intracellular level of ROS, which is known to be related to the processes of cellular activation and death, remained at 0.087 (0.078–0.094) arbitrary units. This did not differ statistically from that in the control culture without TiO_2_-coated titanium samples (0.099 (0.088–0.112) arbitrary units). Nevertheless, a negative correlation was established between the TiO_2_ surface topography and the viable Jurkat T cell count after 24 h of culture (*r_s_* = −0.95; *n* = 9; *p* < 0.0001), which was predominantly demonstrated by an increase in necrosis. A regression analysis showed a linear reduction in the proportion of viable T cells in the culture, whereas the roughness index, *R_a_*, which is associated with the sample topography increased (Figure 4). When exposed to a TiO_2_ surface with an *R_a_* of 2.28–3.7 μm, the median Jurkat T cell survival rate decreased significantly (*P_U_* < 0.05) from 79% to 68% viable cells (compared with control cell growth on plastics, which yielded 82.9% viable cells). 

According to the obtained data (Table 3), short-term exposure of Jurkat T cells to the titanium samples with rough TiO_2_ coatings resulted in a 1.5-fold down-regulation of *hTERT* expression. In addition, the correlation analysis showed a close relationship (*r* = −0.9; *n* = 7; *p* < 0.006) between the reduced *hTERT* expression in Jurkat Т cells and the increase in the average roughness index, *R_a_*, which is associated with the complexity of the TiO_2_ coating topography.

The human T cell lymphoblast-like cells were characterized by a wide range of cell surface markers (Table 4). In the control cell culture, 95% of the cells were CD45RA^+^ naïve (not activated by the antigen) immortalized T cells. The majority of the cells (27–98%) in the 24-h culture displayed the CD3^+^CD4^+^CD71^+^CD45RA^+^ immunophenotype. Markers of cell differentiation, maturation, and death (CD8, CD16, CD56, CD25, and CD95) were observed in 0–2.5% of tumor immune-competent cells (Table 4).

Investigation of the Jurkat T cell cytokine profile revealed the secretion of IL-2, IL-4, IL-8, IL-10, and TNFα, but not IL-1b or IL-6 (Table 5). In this study, we did not use mitogens, cytokines, or chemical activators, many of which usually lead to 100–1000-fold enhancement of cytokine production [32], promote in vitro cell survival, and prevent the toxic influence of irritants.

Notably, the detection of IL-2 in the supernatants (Table 5) was not accompanied by the expression of its receptor (the CD25 antigen) (Table 4). It appears that the IL-2-dependent pathway (the pathway involving the cytokine and the corresponding receptor) does not serve as the key mechanism underlying the autocrine activation of T cell proliferation and the in vitro survival of the tumor clone. However, IL-2-independent activation occurred and was conditioned by cell surface expression of the CD71 proliferation molecule (the transferrin receptor) and the CD95 apoptotic cue (Table 4). As a result, after 24 h of culture, the total karyocyte count increased from 1 × 10^6^/mL to 1.12 (1.1–1.4) × 10^6^/mL, with 82.9% of the T cells surviving. According to Table 4, a subset of the nonadherent Jurkat T cells died through apoptosis (median of 5.2%) and necrosis (median of 8.2%).

The addition of TiO_2_-coated titanium samples to the cell culture changed the morphofunctional state of the human leukemic T lymphoblast-like cells (Table 4 and Table 5). Exposure of the cells to the artificial surface did not increase the expression of the low- molecular-weight isoform of CD45 (CD45RO), which is specific to stimulated T cells. Nevertheless, flow cytofluorometry showed statistically significant decreases (Table 4) in the proportion of nonadherent cells expressing the following T cell differentiation and maturation antigens: CD3, by 0.5%; CD8, by 1.85%; and, CD4, by 2.1%, when compared with the control.

The short-term contact of Jurkat T cells with the TiO_2_ coating was not accompanied by changes in the spontaneous secretion of IL-2, the primary lymphokine produced by the Jurkat cell line. However, the secretion of IL-4, which is also capable of facilitating T cell growth and the generation of cytotoxic Т-lymphocytes [33], was completely abolished (Table 5). 

The reduction in Jurkat T cell survival was not related to the examined TNF superfamily members (CD95 (Fas/APO-1) or TNFα). TNFα secretion and CD95 expression decreased to 90% (*P_U_* < 0.01) and 56% (*P_U_* < 0.002), respectively, of the levels in control cell cultures (Table 4 and Table 5).

**Figure 5:**
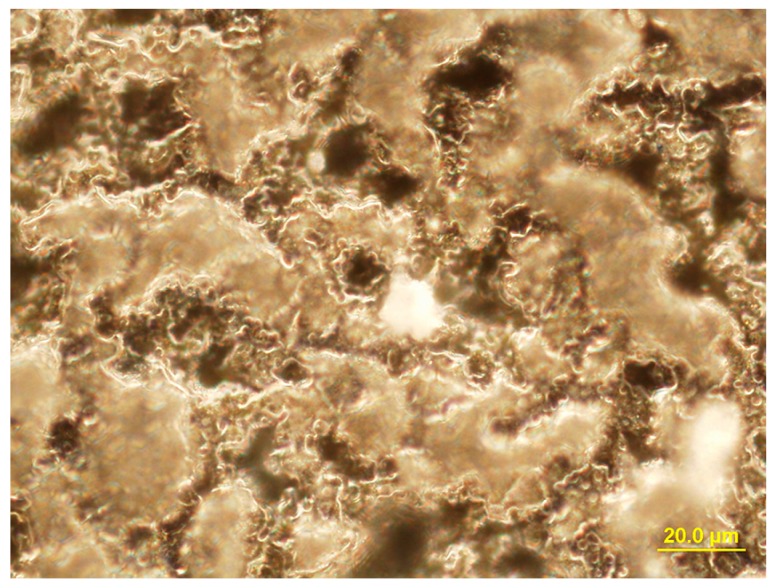
Location of Jurkat T cells on the rough surface of the TiO_2_ coating. Reflecting optical microscopy, dark field. Magnification, 1000×.

The singular molecular genetic hypoergy of the Jurkat T cells induced by the TiO_2_ coating can be accounted for by both direct and indirect effects of the artificial material via the products of its destruction and biodegradation (including nanoparticles). Moreover, a subset of the Jurkat T cells adhered to the TiO_2_ surface (Figure 5).

Changes in surface roughness had a pronounced effect on the morphofunctional parameters of the leukemic T lymphoblast-like cells (Table 6).

The results obtained from the Jurkat T cell cultures (Table 7) showed that titanium dioxide, unlike a Fe_3_O_4_ nanosuspension, as well the TiO_2_ surface itself (see above) at a dosage range of 10–100 MACs had no significant effect on the apoptotic death of leukemic T lymphoblast-like cells. The well-known nano irritant Fe_3_O_4_ depressed apoptotic cell outcome. The intracellular concentrations of ROS did not change in cells exposed to either type of nanoparticles (Table 7).

Conversely, the Jurkat T cells reacted to the nanoscale stimuli by increasing the TNFα concentration in the extracellular medium (up to 22%, regardless of the nanosuspension dose) and, to a lesser extent, the IL-2 concentration (up to 18% at a nanosuspension dose of 100 MACs). It is worth noting that the inverse cytokine effect of the titanium dioxide nanosuspension compared with the influence of the bulk samples (Table 5 and Table 8). In contrast, the suppressive effect of the TiO_2_ nanoparticles on the secretion of IL-4 (11–18% as compared with the control, Table 8) corresponded to that of the TiO_2_ coating (Table 5). Surprisingly, the TiO_2_ nanoparticles at a dose of 10 MACs stimulated higher cytokine levels than the corresponding dose of Fe_3_O_4_ nanoparticles (Table 8).

The obtained results do not allow an unambiguous connection to be established between the effects of the TiO_2_ surface on Jurkat T cells and the nanoscale products of their biodestruction for the following reasons: (1) TiO_2_ nanoparticles influenced the cellular secretion of cytokines ambiguously; (2) tumor Jurkat T cells may have low sensitivity to TiO_2_ or Fe_3_O_4_ nanoparticles at the doses tested despite the marked toxicity of these particles to normal bone marrow cells [34]; (3) high nanoparticle concentrations (1–10 mg/L) can be achieved only through biomechanical impact on the ceramic/metallic TiO_2_ coating, and this is impossible in short-term cell culture; and, (4) TiO_2_ nanoparticles did not trigger intracellular oxidation processes (Table 7).

Measurement of the electrostatic properties of the TiO_2_ coating on the titanium substrate showed a negative surface charge and an average magnitude of potential of V = −147 ± 35 mV (61 runs). The magnitude of the negative surface potential increased with the roughness of the samples (*r* = 0.6; *p* < 0.000001, *n* = 60).

## 4. Discussion

After 24 h of culture on a plastic surface (2D culture), the Jurkat T cells that were used in these experiments expressed a catalytic telomerase subunit with reverse transcriptase function (Table 3). These cells also displayed the predominant CD3^+^CD4^+^CD71^+^CD45RA^+^ immunophenotype (Table 4), which, to a great extent, functionally corresponds to naïve/resting T helper/inductor cells [35,36] with expression of the transferrin receptor (CD71), a T-lymphocyte mitogen [37]. Only 0.55% of the T cells in the 24-h culture displayed the CD45RO isoform of the transmembrane antigen that is expressed in vitro by activated T lymphocytes and/or memory T cells [38,39].

The Jurkat T cells secreted a wide range of immunomodulatory cytokines and chemokines (IL-2, IL-4, IL-8, IL-10, and TNFα) into the extracellular medium, but did not secrete IL-1b or IL-6 (Table 3). The cells displayed pro-inflammatory (IL-2, IL-8, and TNFα) and anti-inflammatory (IL-4 and IL-10) activities [39] IL-2, IL-4 [32], and IL-8 [40] can promote the proliferation and survival of tumor cells by means of autocrine/paracrine signaling pathways. In addition, while IL-1 is considered an autocrine co-stimulator of the growth of T cells [32] and leukemic lymphoblasts [41], including the Jurkat line [42], it is also an endogenous inhibitor of CD95(Fas)-mediated apoptosis [43]. IL-6 also has an anti-apoptotic effect on T cells [36]. The Fas (CD95^+^) transmembrane receptor was observed on only 1% of the Jurkat T cell population (Table 4); therefore, an absence of IL-1b and IL-6 secretion in the Jurkat cell culture (Table 5) could facilitate the cell death processes reported in Table 4. Moreover, IL-10 (Table 5) is capable of inducing anergy in T cells [44], suppressing their proliferation and activation and inhibiting IL-2 expression, as described in [39]. In the absence of co-stimulatory autocrine factors (such as IL-1 and IL-6), the observed imbalance in IL-2 secretion (Table 5) and the absence of its receptor (CD25 antigen, Table 4) can explain the slow in vitro growth of the Jurkat T cells. To a certain extent, these data corroborate the hypothesis regarding the necessity of several ‘survival signals’ for suppressing the genetic cell death program [45].

For a long time, titanium was considered a bio-inert material [9,10] because of the formation of a thin titanium dioxide film on its surface through self-passivation. Micro-arc oxidation of titanium substrates made it possible to increase the average thickness of the TiO_2_ coating to up to 9 μm, implying an increase in corrosion resistance and in the dielectric properties of the sample surface. On average, the metal-ceramic TiO_2_ film did not trigger apoptosis or necrosis of the Jurkat T cells (Table 4). Moreover, exposure of the cell cultures to a potential stimulus was accompanied by the suppression of cell death signaling pathways that were potentiated by the TNFα superfamily receptor CD95 (Fas/APO-1) (Table 4 and Table 5). However, in cells exposed to samples of the TiO_2_ coating with an *R_a_* greater than 2.2 μm, the morphofunctional inactivation of the Jurkat T cells was accompanied by a progressive decrease in the viability of the cells in the culture (Figure 4) in the context of complete suppression of IL-4-dependent mechanisms of T cell survival (Table 5). The underlying molecular genetic mechanism could involve the observed 1.5-fold down-regulation of *hTERT* expression following exposure (Table 3), which correlated with increased TiO_2_ surface roughness. Telomerase forms a ribonucleic acid complex that is crucial for supporting cell proliferation; it becomes increasingly active in most tumor cells, including Jurkat T cells [46,47]. Expression of the telomerase gene (*hTERT*) contributes to the viability of Jurkat T cells [46] and results in elevated production of cytokines [48]. The data obtained in the present study suggest a certain toxic effect of the TiO_2_ coating that is mediated by inhibition of the genetic (preserving the length of telomeric DNA sequences) and secretory (IL-4) mechanisms supporting the proliferation of tumor Jurkat T cells. 

Taken together, the observed effects of the TiO_2_ nanosuspensions (Table 7 and Table 8) do not allow for it to be concluded that nanoparticles are a proven mediator of the negative cellular and molecular effects of the TiO_2_ coating on cell viability. Some researchers [7] argue that titanium oxide nanoparticles, unlike the ions and oxides of other metals, do not display cytotoxicity against Jurkat T cells [1,6]. Other reports claim that TiO_2_ nanoparticles are capable of causing in vitro damage to and apoptosis of lymphocytes [49] and leukemic cells, not through their endocytosis but through direct contact with the cytoplasmic cell membrane [50]. Nanoparticles can be adsorbed on the surface of Jurkat cells [51].

In the present study, the interaction of the Jurkat T cells with the topography of TiO_2_ particles, but not with the products of their destruction, appears to cause the observed decrease in cell viability and functional activity. A close connection between the *R_a_* and the genetic and molecular features of Jurkat T cells was established (Figure 4 and Table 6). The correlations of *R_a_* with the CD antigen profile and the proportions of apoptotic and necrotic cells (Table 6) suggest that the cell surface is a target of the impact of the physical properties of the TiO_2_ surface. However, the mechanisms underlying the down-regulation of *hTERT* expression associated with TiO_2_ surface roughness have not yet been established. 

The process of necrosis begins with the loss of membrane integrity. Membrane channels are sensitive to the topography of biomaterials [52]. For instance, the entry of extracellular calcium and the release of calcium from intracellular stores are triggers of T cell activation [53] and/or programmed cell death [54]. However, non-activated Jurkat T cells are capable of adhering to the TiO_2_ surface, primarily at zero-charged segments, through the electrostatic interaction of negatively charged glycoproteins on their surface membranes (zeta potential) [5]. The micro-arc TiO_2_ coating has a negative charge and a surface electrostatic potential that increases linearly with the surface roughness. A subset of T lymphoblast-like cells adheres to the TiO_2_ surface (Figure 4). These cells are exposed to the surface charge carriers. The electric field of the surface appears to be a physical factor that is capable of modulating the activity of intracellular signaling pathways and molecules. The lifted electrode of the electric potential measuring device registers the electric field at a distance of 500 μm (approximately 50 cell diameters) from the measured surface [28]. This suggests that, over a period of 24 h, the electrostatic potential of the TiO_2_ sample coating covering 77% of the well surface in the culture plate impacts the majority of the Jurkat T cell population. A favorable impact of negatively charged calcium phosphate coatings (with a comparable surface roughness) on the differentiation and maturation of normal human stromal cells has been previously described [55]. A change in the value and sign of the transmembrane potential that is related to the zeta potential would significantly affect cell fate [56].

The obtained results suggest that the roughness of the TiO_2_ dielectric surface induces an electrostatic potential that is capable of altering the molecular genetic features and viability of immortalized human leukemic T lymphoblast-like cells through mechanisms unrelated to ROS generation. The functional hypoergy of immune-competent cells can explain the high survival rate of titanium implants in the human body and can impact the choice of materials for endoprosthetics and osteosynthesis in patients suffering from hematopoietic and lymphoid malignancies.

## 5. Conclusions

When compared with other surface modification techniques, MAO is one of the most applicable methods to deposit a bioceramic layer on Ti and its alloys and to improve the biological properties of titanium [57]. Titanium substrates with TiO_2_ coatings are widely used in orthopedics, traumatology, dental implantology, and oral surgery. This study established down-regulating expression of membrane antigens that are indicative of the activation, differentiation, and maturation of tumor Jurkat T cells (CD3, CD4, CD8, and CD95) and reducing the secretion of IL-4 and TNFα. The inactivation of Jurkat T cells caused by short-term exposure to the rough TiO_2_ coating is not related to the generation of intracellular ROS and is not mediated by TiO_2_ nanoparticles released from the biodegradation of titanium implants and endoprosthetics. Nevertheless, the inhibitory effect of a TiO_2_ *R_a_* of 2.2–3.7 μm on the viability of Jurkat T cells primarily occurred through a progressive increase in the proportion of necrotic cells in the cell population. In turn, increased *R_a_* was accompanied by an increase in the magnitude of the negative electrostatic potential of the TiO_2_ surface.

Thus, rough titanium oxide coating (rTOC) inhibits Jurkat T cell survival in 24-h culture. rTOC causes in vitro morphofunctional inactivation of Jurkat T cells. The impact of rTOC is not related to intracellular ROS generation. Jurkat T cells have low sensitivity to TiO_2_ nanoparticles. Negative surface charge induced by rTOC might mediate its cellular effects.

We suggest that the roughness of the dielectric TiO_2_ coating induces a negative charge that is likely capable of structural (down-regulation of antigen expression) and functional (down-regulation of *hTERT* expression and cytokine secretion) regulation of tumor immune cells by means of biological mechanisms and metabolic pathways unrelated to ROS generation. The in vitro inactivation of Jurkat T cells caused by a rough TiO_2_ coating suggests that it is important to study the effects of such coatings on other tumor cell lines and to identify intracellular signaling pathways that are susceptible to physicochemical factors. Hereafter, this may be useful for replacement surgery applications of rough TiO_2_ implants in cancer patients.

## Figures and Tables

**Table 1:** Sequences and probes used for polymerase chain reaction (PCR) to measure *hTERT* expression in Jurkat T cells.

Gene	Primers and Probes	Amplicon Mass
*hTERT*	Forward: 5′-ACCGTCTGCGTGAGGAGATC-3′ Reverse: 5′-CCGGTAGAAAAAGAGCCTGTTC-3′	131 base pairs
18S rRNA	Forward: 5′-CTGAGAAACGGCTACCACATC-3′ Reverse: 5′-GCCTCGAAAGAGTCCTGTATTG-3′	153 base pairs

**Table 2:** Monoclonal antibody panel used for Jurkat T cell analysis.

Studied Subpopulation	Fluorochromes and Labeled Monoclonal Antibodies				
	PE	FITC	VioBlue	PE	APC
Jurkat T cells	Propidium iodide	Annexin V	CD3	CD56	CD16
				CD4	CD8
				CD71	CD95
				CD45RA	CD45RO
				-	CD25

**Table 3:** *hTERT* expression levels in Jurkat T cells after 24 h of culture in the presence of a model matrix consisting of a TiO_2_ coating on a titanium substrate, Me (Q_1_–Q_3_).

Group Studied (*n* = 7)	*hTERT*, a.u.
Control cell culture	9.81 (8.90–11.18)
Cell culture in contact with the TiO_2_-coated titanium samples	6.00 (4.48–6.95) *

Note: Here and below, *n* = the number of observations (samples) in each group; * = statistical significance (*p* < 0.05) in comparison with the control according to the Mann-Whitney U-test; a.u. = arbitrary units.

**Table 4:** Membrane molecular markers and death indices of Jurkat T cells after 24 h of culture in the presence of the TiO_2_ coating Me (Q_1_–Q_3_).

Dead or Necrotic Cells, %		Cells Expressing Specific Surface Markers, %									
Apoptosis	Necrosis	CD3	CD4	CD8	CD71	CD45RO	CD45RA	CD16	CD56	CD25	CD95
Cells cultured on plastic surface (control), *n* = 15											
5.2 (5.2–8.9)	8.2 (8.1–14.2)	98 (97.9–98.85)	27.5 (26.54–28.43)	2.16 (1.9–3.0)	94.0 (93.5–95.0)	0.55 (0.49–0.60)	94.9 (94.0–95.22)	2.46 (2.34–2.83)	2.5 (2.3–2.97)	0 (0–0.16)	1.0 (0.9–1.2)
Cells in contact with the TiO_2_-coated titanium samples, *n* = 15											
5.9 (5.6–6.9)	15.3 (8.2–19.3)	97.5 (97–98) * <0.03	25.4 (24.76–26.70) * <0.002	0.31 (0.2–0.43) * <0.000003	94.1 (93.49–95.0)	0.50 (0.41–0.50)	94.0 (93.15–94.3)	2.33 (2.17–2.34)	2.36 (2.22–2.80)	0 (0–0.12)	0.56 (0.3–0.6) * <0.002

* = statistical significance (*p* < 0.05) in comparison with the control according to the Mann-Whitney U-test.

**Table 5:** Cytokine concentrations (pg/mL) in the supernatants of Jurkat T cells after 24 h of culture in the presence of a TiO_2_ coating, Me (Q_1_–Q_3_).

Cytokine Concentration						
IL-1b	IL-2	IL-4	IL-6	IL-8	IL-10	TNFα
Control cell culture on plastic surface, *n* = 12						
0 (0–0.23)	5.67 (5.46–5.98)	1.10 (0.74–1.24)	0	11.12 (9.18–12.59)	6.10 (5.40–6.96)	14.95 (13.97–15.51)
Cell culture in contact with the TiO_2_-coated titanium samples, *n* = 12						
0 (0–0.10)	6.10 (3.30–8.10)	0 (0–0.79) * <0.007	0	12.78 (11.71–13.22)	7.80 (4.87–9.86)	13.48 (8.0–14.15) * <0.01

* = statistical significance (*p* < 0.05) in comparison with the control according to the Mann-Whitney U-test.

**Table 6:** Spearman’s correlation coefficient between the *R_a_* of the TiO_2_ coating and the morphofunctional parameters of the Jurkat T cell cultures.

Index	% Viable Cells	% Apoptotic Cells	% Necrotic Cells	CD8	CD56	IL-2	IL-4	IL-10	TNFa
*R_a_*	−0.95 *n* = 9 0.0001	−0.69 *n* = 9 0.042	0.95 *n* = 9 0.0001	0.71 *n* = 12 0.004	−0.95 *n* = 12 0.0001	−0.95 *n* = 12 0.000003	−0.69 *n* = 12 0.013	−0.92 *n* = 12 0.00003	0.68 *n* = 12 0.015

**Table 7:** Proportions of apoptotic Jurkat T cells and intracellular levels of ROS after 24 h of culture with nanoparticle suspensions, Me (Q_1_–Q_3_).

No.	Group	Results	
		Number of Apoptotic Cells, %	ROS, a.u.
1	Cells cultured on plastic surface without nanoparticle suspension, *n* = 6	5.87 (5.32–6.99)	0.130 (0.123–0.150)
Concentration of TiO_2_ nanoparticle samples, *n* = 3			
2	100 MACs	4.82 (4.24–8.50)	0.131 (0.129–0.194)
3	10 MACs	4.76 (3.65–6.39)	0.139 (0.130–0.162)
Concentration of Fe_3_O_4_ nanoparticle samples, *n* = 3			
4	10 MACs	4.39 (3.05–5.10) P1 < 0.02	0.123 (0.103–0.150)

Note: MACs = maximum allowable concentrations; a.u. = arbitrary units; P1 significant difference compared with the corresponding group according to the Mann-Whitney U-test.

**Table 8:** Cytokine levels (pg/mL) in the culture medium after 24 h of culture of Jurkat T cells with nanoparticle suspensions, Me (Q_1_–Q_3_).

No	Group	Cytokine Concentration		
		TNFα	IL-2	IL-4
1	Cells cultured on plastic surface without nanoparticle samples, *n* = 6	27.41 (26.62–28.17)	4.02 (3.97–4.04)	4.14 (4.06–4.24)
Concentration of TiO_2_ nanoparticle samples, *n* = 3				
2	100 MACs	33.52 (32.77–35.0) P1 < 0.05	4.73 (4.61–5.0) P1 < 0.05	3.67 (3.60–3.67) P1 < 0.045
3	10 MACs	33.51 (33.17–35.49) P1, P4 < 0.05	3.61 (3.56–4.55)	3.40 (3.27–3.60) P1, P4 < 0.045
Concentration of Fe_3_O_4_ nanoparticle samples, *n* = 3				
4	10 MACs	26.62 (24.72–27.34)	4.23 (4.0–4.29)	4.39 (4.10–4.52)

Note: P1, P4 = statistically significant differences compared with the corresponding group according to the Mann-Whitney U-test.
